# Exosomes in Pathogen Infections: A Bridge to Deliver Molecules and Link Functions

**DOI:** 10.3389/fimmu.2018.00090

**Published:** 2018-02-12

**Authors:** Wenchao Zhang, Xiaofeng Jiang, Jinghui Bao, Yi Wang, Huixing Liu, Lijun Tang

**Affiliations:** ^1^School of Life Science, Central South University, Changsha, China; ^2^XiangYa School of Medicine, Central South University, Changsha, China

**Keywords:** exosome, pathogen, infection, immune regulation, transmit carrier, apoptosis

## Abstract

Exosomes are extracellular vesicles derived from cell endocytosis which act as transmitters between cells. They are composed of proteins, lipids, and RNAs through which they participate in cellular crosstalk. Consequently, they play an important role in health and disease. Our view is that exosomes exert a bidirectional regulatory effect on pathogen infections by delivering their content. First, exosomes containing proteins and RNAs derived from pathogens can promote infections in three ways: (1) mediating further infection by transmitting pathogen-related molecules; (2) participating in the immune escape of pathogens; and (3) inhibiting immune responses by favoring immune cell apoptosis. Second, exosomes play anti-infection roles through: (1) inhibiting pathogen proliferation and infection directly; (2) inducing immune responses such as those related to the function of monocyte-macrophages, NK cells, T cells, and B cells. We believe that exosomes act as “bridges” during pathogen infections through the mechanisms mentioned above. The purpose of this review is to describe present findings regarding exosomes and pathogen infections, and highlight their enormous potential in clinical diagnosis and treatment. We discuss two opposite aspects: infection and anti-infection, and we hypothesize a balance between them. At the same time, we elaborate on the role of exosomes in immune regulation.

## Introduction: What are Exosomes?

### History

Exosomes are extracellular vesicles (EVs) formed intracellularly by a process of endosome membrane invagination which generates multivesicular bodies (MVBs) containing intraluminal vesicles (ILVs) (1). Canonical exosomes measure 50–100 nm, have a density of 1.13–1.19 g/ml in sucrose density gradients and present a cup-shaped morphology on examination by transmission electron microscopy (2). These membrane vesicles were initially found in rat reticulocytes (3). Compared with cell membranes, exosome membranes are enriched in lipids such as cholesterol and sphingomyelin and in lipid-raft-associated proteins (4). The term “exosomes” for these EVs of endosomal origin was first proposed in 1987 (5). Since then, interest in exosomes has consistently grown, with an ever-increasing number of studies focusing on the function and application of exosomes in pathogen infections and other pathological conditions.

### Biogenesis

According to present knowledge, the biogenesis of an exosome consists of four stages: initiation, endocytosis, MVB formation, and secretion (6).

At first, membrane-associated molecules, such as nucleic acids, proteins and others, are internalized *via* endocytic vesicles formed by invagination of the plasma membrane. Endocytic vesicles then fuse with early endosomes and deliver their content to them. Early endosomes mature into late endosomes characterized by the presence of ILVs in their lumen, reason for which they are also called MVBs (7, 8). The main fate of MVBs is to fuse with lysosomes, where their content is degraded. Another possibility is for MVBs to merge with the plasma membrane, therefore, releasing its ILVs into the extracellular space, where they are called exosomes (9). Therefore, the composition of exosomes is expected to reflect to some extent the composition of MVBs. For instance, proteins of the endosomal sorting complex required for transport (ESCRT) and CD63 are associated with MVBs and have also been found in exosomes (10). Due to the complexity of endocytic pathways, the mechanisms regulating exosome release have not been well elucidated to date.

### Isolation and Detection

With the development of technology, more and more strategies were continually applied for detecting and isolating exosomes, promoting the exploration of exosomes. Among these techniques, we can mention transmission microscopy, ultracentrifugation, density-gradient separation, immunoaffinity capture (11), and microfluidic systems (12). Based on the small size and low density of exosomes, ultracentrifugation is the most developed and commonly used method for exosome isolation. This technique employs an exceedingly high centrifugal force, which can reach 100,000 g, to precipitate subcellular components or even macromolecules. However, it is very time-consuming and the exosome purity achieved is poor (13). As technology improves, new separation techniques have emerged such as sequential filtration (14). Considering the importance of exosomes, a low-cost, hypersensitive, and simple detection method is desirable. Relatively new, stochastic techniques for exosome detection are photoactivated localization microscopy (PALM) or stochastic optical reconstruction microscopy (STORM). PALM and STORM are based on single-molecule localization to track exosomes, which can be observed down to the nanometric level and allow the visualization of intracellularly incorporated exosomes (15).

## Discrepant Expression of Exosomes from Infected and Uninfected Cells

Exosomes play an important role during the biological processes following pathogen infections, with changes in exosome quantity, content, and membrane structure being detected. In this section, a brief description of these changes is provided, whereas details on mechanisms and functions will be discussed in later sections.

### Alterations in the Numbers of Exosomes Generated

Due to the altered cellular activity of infected cells and the utilization of endocytic pathways of host cells by pathogenic organisms, the number of exosomes generated by host cells may change in relationship with the transmission of infection. For instance, it has been shown that patients infected with Plasmodium presenting symptoms for >6 days exhibit an increase of 20–30% in platelet-originated exosomes. Of note, the levels of plasma exosomes decreased at least 20% after 21 days of treatment (16). In a rotavirus (RV) study, the culture media from RV-infected cells contained a higher amount of heat shock cognate protein 70, TGF-β1, and other exosome proteins than those from control-treated cells, suggesting that RV infection of human intestinal epithelial cells increases the release of EVs (17). The presence of pathogens can also drive exosome production. In a study of *Mycobacterium bovis* Bacille Calmette–Guerin (*M.bovis* BCG) infection in mice, the kinetics of bacterial load showed an initial increase that peaked at day 10 followed by a gradual decline through to day 60. Interestingly, the exosome concentration in serum showed similar kinetics, with a peak value approximately 100-fold higher compared with a normal, uninfected condition. This suggested that infection induces exosome secretion and this is correlated with the bacterial burden (18). Recent studies have speculated on the mechanisms by which the number of exosomes derived from different cells could be affected during infection. First, the intracellular synthesis of exosomal marker proteins increases in association with infection. Second, pathogens seem to promote molecule assemblage and secretion activity in infected cells. For example, the presence of the viral matrix protein viral protein 40 (VP40) in ebola virus (EBOV)-infected cells is known to induce an upregulation of the exosomal markers CD63, apoptosis-linked-gene-2 product-interacting protein X (Alix) and Endosomal Sorting Complex Required for Transport machinery-II proteins, indicating that exosomal biogenesis is activated during EBOV infection (19). In addition, unlike other pathogens, parasites themselves can secrete exosomes for intercommunication purposes and, therefore, exosomes are found increased in body fluids from parasite-infected organisms.

### Changes in Exosome Membrane Structure

Exosomes are vesicle structures with an external membrane consisting of a phospholipid bilayer with which proteins, carbohydrates, lipids, and nucleic acids are associated (20). The structure of exosomal membranes often changes as a consequence of infections, including alterations in the quantity of structural proteins and lipids and even spatial configuration inversions. The protein content of exosomes has been shown to be modified under pathological or stress status (21). Simbari et al. have suggested that, after nematode infection, an increase in plasmalogen in exosome membranes is counteracting the diminished levels of other lipids, such as cholesterol and sphingomyelin (22). Diaz et al. demonstrated that 26 membrane-associated proteins were significantly more abundant in exosomes from *Mycobacterium tuberculosis* (*M.tb*)-infected macrophages (23). In exosomes from the urine of leptospira-infected rats, the membrane protein alanyl aminopeptidase (CD13) was significantly increased (24).

### Alterations of Exosome Content

A body of research confirms that infections with pathogenic organisms lead to significant changes in exosome content, such as protein kinase G in *M.tb* infections (25), abundant larval transcript protein in infections with the filarial parasite *B. malayi* (26), cellular prion protein (PrP C) in prion disease and Alzheimer disease (27), lipopolysaccharide (LPS) in infections with Gram-negative bacteria (28), and nucleic acids [EBV-miR-BART3 and EBV-miR-BHRF1-1 in Epstein–Barr virus (EBV) infections (29), miRNA-200 in *H. polygyrus* infections (30), miRNA-155, and miRNA-132 in *M.tb* infections (31)]. Additional information is listed below in Table 1.

**Table 1:** Alterations of exosome content triggered by infections.

Exosomal contents	Secreting cells	Changing trend	Infection involved	Function	Reference
EBOV VP40 (structural protein)	EBOV-infected cells	Upregulation	EBOV infection	Modulating RNAi components in recipient immune cells, ultimately resulting in cell death	(19)

ALT-2 protein	Larva of the filarial parasite *B. malayi*	Upregulation	Filariasis	Inducing the signaling proteins GATA-3 and SOCS-1, which act to induce type 2 responses and dampen IFN-γ-dependent inflammatory signals in the cell	(26)

Cellular prion protein (PrP C)	Neuroblastoma cells	Upregulation	Prion disease; Alzheimer disease	Accelerating fibrillization of amyloid beta and reducing neurotoxic effects imparted by oligomeric Aβ	(27)

LPS	Gram-negative bacteria	Upregulation	Gram-negative bacteria infection	Promoting caspase-11 activation and host defense against bacterial infection and pathogenesis of sepsis	(28)

Bacterial pore forming α-toxin	*Staphylococcus aureus*	Upregulation	*S. aureus* infection	Allowing for delivery of bacterial virulence factors to distant cells	(42)

Lethal toxin virulence factor	*B. anthracis*-infected cells	Upregulation	*B. anthracis* infection	Allowing for the delivery of LT to cells at sites distal to infection	(43)

CagA	CagA-expressing gastric epithelial cells	Upregulation	*Helicobacter pylori* virulence infection	Developing extra-gastric disorders associated with CagA-positive *H. pylori* infection	(44)

Viral transactivator Tax	HTLV-1-infected T-cell lines	Upregulation	HTLV-1 infection	Activating transcription of target cells	(45)

Mature virions	HHV-6-infected cells	Upregulation	HHV-6-infection	Spreading infection faster through exosomes	(47)

Serum resistance-associated protein	*T. brucei*	Upregulation	*T. brucei*	Allowing evasion from human innate immunity	(50)

Immunogenic variant surface glycoprotein	*T. brucei*	Upregulation	*T. brucei*	Altering the physical properties of the erythrocyte membrane and causing clearance of infected erythrocytes by macrophages in the liver and spleen	(50)

VPS4B and ALIX protein	HAV-infected cells	Upregulation	HAV infection	Facilitating escape from neutralizing antibodies and probably promoting virus spread	(53)

HBx	HBV-infected hepatocytes	Upregulation	HBV infection	Resulting in decrease of intracellular APOBEC3G protein level, therefore, enhancing infection	(62)

EBV-miR-BART3 and EBV-miR-BHRF1-1	EBV-transformed lymphoblastoid cell line	Upregulation	EBV-infection	Indicated as crucial in the crosstalk between EBV and the host microenvironment	(29)

miRNA-200, miR-16, miR-71	Nematode parasites	Upregulation	*H. polygyrus* infection	Suppressing Type 2 responses and then suppressing innate immunity responses	(30)

miR-21, miR-29a	HBV-infected hepatocytes	Upregulation	HBV infection	Suppressing IL-12p35 mRNA expression to counteract host innate immune responses	(61)

*aatk, slc7a1 and cdkal* (mRNAs encoding HIV-1 Nef protein)	Human monocytes	Upregulation	HIV-1	Involved in large-scale bystander cell death of uninfected CD4^+^ T cells and dysregulation of fatty acid metabolism	(64)

let-7f, miR-145, miR-199a, and miR-221	Umbilical mesenchymal stem cells	Upregulation	HCV (hepatocyte virus) infection	Targeting specific cellular factors or directly binding to viral genomes to block productive HCV replication	(68)

HCV ss-RNA (associated with miR-122 and Ago-2)	HCV-infected hepatocytes	Upregulation	HCV infection	Increasing inflammation in the liver and leading to liver fibrosis	(74)

## The Role of Exosomes in Homeostasis

Exosomes act as modulators to maintain the homeostasis of our body, as shown by researchers who studied how exosomes participate in a number of physiological events at the level of molecules, tissues, and organs. It is particularly relevant to examine the role of exosomes in the immune system, based on the fact that exosomes can serve as messengers between different immune cells. For example, exosomes from mesenchymal stem cells (MSCs) can activate toll-like receptor (TLR) signaling. Further studies demonstrated that MSC exosomes induced a subdued pro-inflammatory and an enhanced anti-inflammatory IL10 expression. Treg polarization was induced when CD4^+^ T cells were incubated at a 1:1,000 ratio with THP-1 cells that had been treated with MSC exosomes (32). It has been also observed that exosomes released by B lymphocytes are able to stimulate specific CD4^+^ T-cell clones *in vitro*, revealing a role for exosomes in peptide-MHC class ΙΙ complexes presentation (33). Other investigations have found that CD63^+^ exosomes transfer miRNAs unidirectionally from T-cells to APCs during immune synapse formation, resulting in gene expression changes in the recipient cells (34). Chaperones, such as heat shock protein 40 (HSP40) and heat shock protein 70 (HSP70), can be delivered *via* exosomes to target cells, mediating a non-cell-autonomous maintenance of protein homeostasis. It is speculated that this could be part of a mechanism compensating for a disequilibrium in the stress response of different cells of the same organism (35).

Exosomes also crucially influence the transmission of amyloid β-protein (Aβ) within cerebrospinal fluid (36, 37), sending and receiving biological messages to/from cardiomyocytes (CMs) (38), the expression of muscle genes and modulation of muscle differentiation (39), melanin synthesis enhancement (40), etc.

## The Role of Exosomes in Pathogen Infections

Diseases caused by bacteria, parasites, or viruses (e.g., malaria, tuberculosis, and acquired immune deficiency syndrome) affect over one hundred million people worldwide (41). During the past decades, knowledge about exosomes has developed in a variety of directions and more particularly regarding pathogen infections. Exosomes can either accelerate or inhibit the process of infection. In both cases, exosomes make possible connections between host cells or between pathogens and host cells.

### Exosomes Act as a Bridge for the Delivery of Molecules and the Connection of Functions

Exosomes play a crucial role in infections as carriers of substances of pathogen origin. They can directly transmit pathogen-related molecules and also indirectly influence the infection progress through modulating the processes, such as immune evasion and apoptosis. We will detail the different ways in which they exert their action.

#### Mediating Further Infection through the Transmission of Pathogen-Related Molecules

It is currently believed that exosomes can act as transmitters of pathogen-related molecules that help spread the infection in body microenvironments. Regarding bacteria, *Staphylococcus aureus*-derived exosomes have been reported to contain the bacterial pore forming molecule α-toxin, therefore delivering this bacterial virulence factor to distant cells (42). Similarly, exosomes from *Bacillus anthracis*-infected cells have been observed to transport the lethal toxin virulence factor to sites distal to the infection (43). The function of exosomes in *Helicobacter pilori* (*H. pylori*) infection has also been studied. Exosomes secreted from cytotoxin-associated gene A (CagA)-expressing gastric epithelial cells enter the circulation and deliver CagA, a virulence factor, to distant organs and tissues. The delivery of CagA has been proposed to be involved in the extragastric disorders commonly associated with H. pylori infection (44). Concerning viruses, their main objective is to favor the expression of pathogenic genes. During viral infections, exosomes are vehicles of viral components, such as proteins, mRNAs, and microRNAs which are carried to target cells. For instance, exosomes produced by human T-cell leukemia virus-1 (HTLV-1)-infected T-cell lines deliver the viral transactivator Tax which can activate transcription in target cells (45). It has been found that exosomes derived from human immunodeficiency virus-1 (HIV-1) and HTLV-1-infected cells contain proteins of viral and cellular origin that inhibit target cell migration as well as dsRNA/ssRNA which can increase nuclear gene expression and promote infection (46). Exosomes from cells infected with human herpesvirus 6 (HHV-6) contain mature virions; therefore, they help spread infection more efficiently (47). Exosomes have also been found associated with HIV-1 transactivator of transcription (TAT). TAT was able to cause neurite shortening and neuron death (48). Prions are proteinaceous infectious particles that can cause transmissible spongiform encephalopathies (TSEs) in mammals. The yeast *Saccharomyces cerevisiae* can harbor several prions, therefore, constituting a useful investigation model. The prototype yeast prion contains the translation termination factor Sup35. It has been demonstrated that cytosolic Sup35 NM prions are packaged into exosomes. These exosomes are able to transmit the prion phenotype to neighboring cells (49). Thus, EVs disseminate epigenetic information through protein transfer. Recipient cells can be changed by exosomes at the level of protein or nucleic acid, leading to pathological consequences in cells or tissues.

Exosomes from parasites can be involved in virulence and cytotoxicity. For example, nanotube-derived EVs from bloodstream forms of *Trypanosoma brucei* (*T. brucei*) have been shown to fuse with host erythrocyte membranes, with fusion being mediated by an unidentified EV surface protein. Fusion results in the transfer of lipids and parasite-specific antigens, including the immunogenic variant surface glycoprotein, to the erythrocyte surface. This interaction alters the physical properties of the erythrocyte membrane and may cause clearance of infected erythrocytes by macrophages in the liver and spleen (50). It has been observed that *Toxoplasma gondii* (*T. gondii*) can alter host cell (L6 cells) proliferation mechanisms by increasing the number of cells in S phase, and that exosomes enhance this effect by transferring molecules to uninfected neighboring cells (51). Another interesting finding is that exosomes derived from mature red blood cell (RBC) during malaria infection carry a functional RNA-induced silencing complex with Argonaute 2 which is able to specifically silence gene expression in endothelial cells so as to alter their barrier property, thus supporting malarial infection (52).

**Figure 1:**
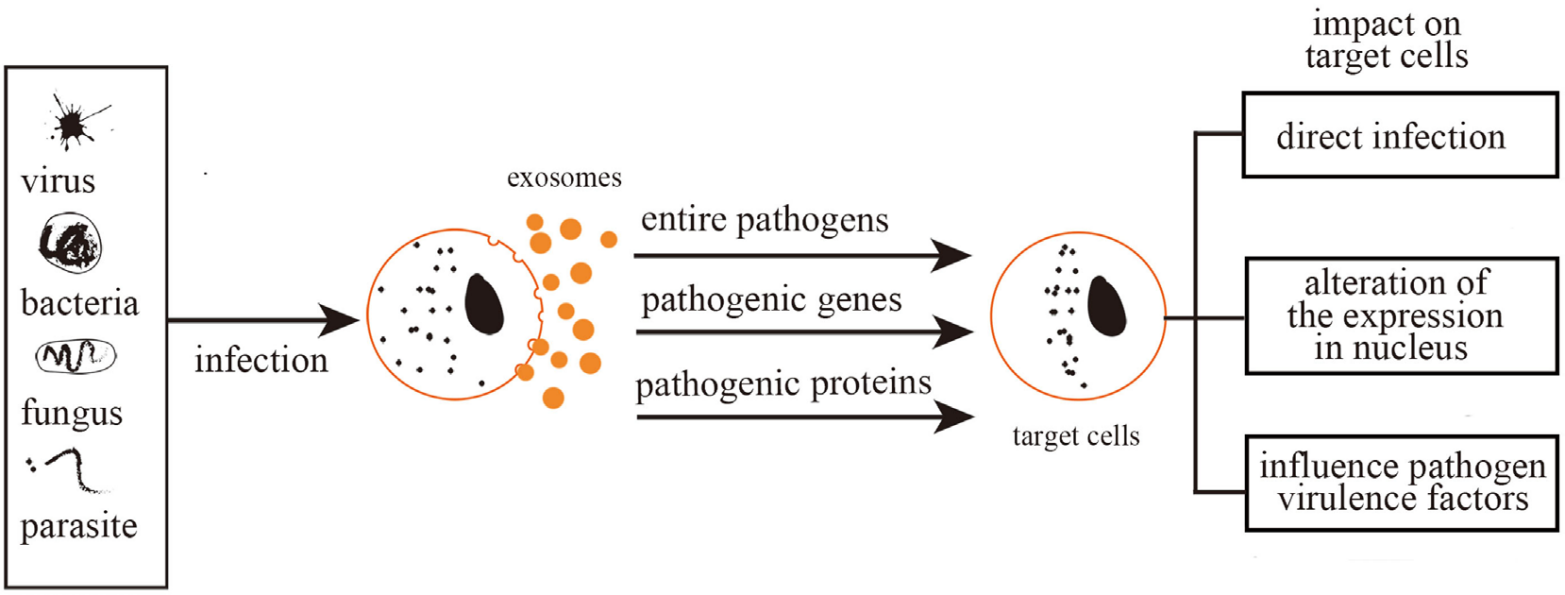
Exosomes mediate further infection. Exosomes mediate further infection through transferring pathogen-related molecules (pathogenic genes and proteins) or even the entire pathogens. Therefore, exosomes can be either directly infectious, alter nuclear gene expression, or mediate toxic reactions.

Altogether, exosomes act as agents for the packaging of complete pathogens and/or related molecules (proteins, nucleic acids, lipids). The delivery of exosome contents to cells mediates the continuation and enhancement of infection processes (Figure 1).

#### Participating in the Immune Escape of Pathogens

Some pathogens can escape the host immune system with the help of exosomes and this favors their spread. For example, release of hepatitis A virus (HAV) from cells after being anchored within host cell membranes protects the virion from neutralization by antibodies. Therefore, an exosomal route avoids contact of the virus with neutralizing antibodies and allows a better virus spread. During this process, the proteins vacuolar protein sorting 4 homolog B (VPS4B) and ALIX play an important role (53). It has also been found that exosomes from virus-infected cells can spread miRNAs of non-host origin making them undetectable to the host immune system (54). In summary, the packaging of pathogen-related molecules makes good sense concerning immune evasion. The present section provides some conclusions and discusses future research directed at suppressing the spread of pathogens by interfering with exosomal pathways.

Similarly, recent studies found that hepatic exosomes can help transmit hepatitis C virus (HCV) infection *in vitro* due to HCV being protected from antibody neutralization (55). An interesting investigation found hepatitis E virus (HEV) RNA-containing particles in an exosome fraction. These particles are infectious and cannot be neutralized by anti-HEV antibodies (56). Therefore, modifications in the RNA packaged within exosomes can be assumed to have occurred, with the specific mechanism still unexplored.

**Figure 2:**
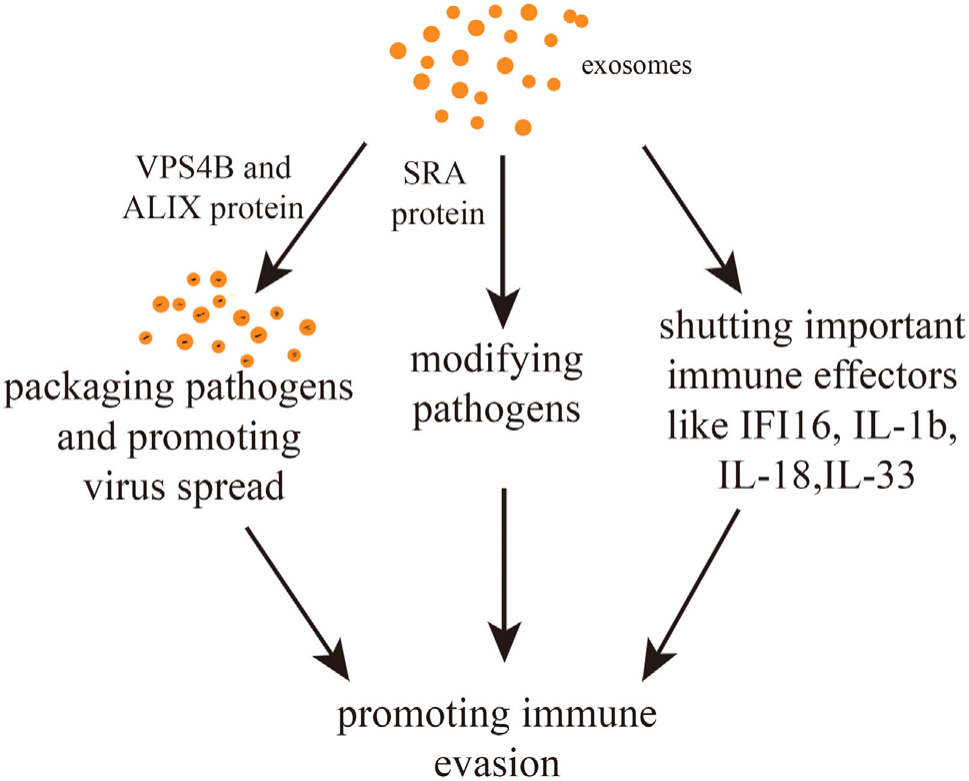
Exosome-mediated immune evasion pathways. Exosomes can package pathogens, modifying them or shutting down important immune effectors. The proteins vacuolar protein sorting 4 homolog B (VPS4B) and ALIX may mediate the spread of packaged pathogens. The protein serum resistance-associated protein (SRA) mediates the alteration of pathogens.

Interestingly, communication between parasites and between parasites and host cells can help immune evasion. In fact, *T.b*. rhodesiense-derived EVs can transfer the serum resistance-associated protein (SRA) to *T.b. brucei*. SRA is needed to circumvent the action of host lytic factors, therefore, conferring the ability to evade innate immunity (50). Other studies have uncovered the important role of exosomes in suppressing immune-related molecules or cells with the consequence of halting the spread of pathogens. For example, EBV has been postulated to escape immune responses by sequestering immune effectors, such as caspase-1, interleukin 1b (IL-1b), IL-18, and IL-33, in exosomes that are continuously secreted (57). Exosomes derived from HIV-1-infected cells enable HIV-1 replication within resting human primary CD4^+^ T cells (58). Equally, exosomes secreted by nematode parasites can suppress an innate type 2 response *in vivo* by downregulating IL-33 release (30). Furthermore, it has been found that *Trypanosoma cruzi*-derived exosomes lead to an increased secretion of IL-4 and IL-10 and a diminished inducible nitric oxide synthase expression in CD4^+^ T cells and macrophages. This induces a Th2 immune response polarization (59, 60). Exosomes derived from hepatitis B virus (HBV)-infected hepatocytes transport miR-21, miR-29a, and other miRs with immunoregulatory functions to THP-1 macrophages, which results in a downregulation of *IL-12p35* mRNA expression in turn leading to a constrained host innate immune response (61). Similarly, it was found that hepatitis B viral X protein (HBx), a small non-structural X protein encoded by HBV, can enhance the externalization of apolipoprotein B mRNA-editing catalytic polypeptide-like protein 3G (APOBEC3G) within exosomes. Since APOBEC3G is an inhibitor of HBV replication, a decrease in its intracellular level favors the infection (62). In summary, there are mainly three immune evasion pathways. First, pathogens packaged within exosomes might not be detected by immune cells. Second, pathogens or pathogen-related substances can be modified within exosomes. Third, some immunity-related molecules can be suppressed by exosomes (Figure 2).

#### Promoting Immune Cell Apoptosis

Inhibiting immune responses is an effective way to favor pathogen spreading and exosomes can act as messengers in this process. A number of investigations indicate that exosomes released by infected cells mediate the inhibition of immune responses mainly accelerating the apoptosis of immune cells. Apoptosis is a process of programmed cell death that occurs in multicellular organisms and can also be induced by alterations in the microenvironment. Apoptotic cells show membrane blebbing, shrinkage, nuclear fragmentation, chromatin condensation, chromosomal DNA fragmentation, and global mRNA decay. Exosomes released from virus-infected cells have been shown to contain viral RNA and proteins that trigger the induction of apoptosis on interaction with T- and monocytic cells. Such apoptosis is regulated by poly ADP-ribose polymerase 1 and caspase 3 (63). Exosomes secreted from Nef-expressing U937 monocytic cells can enter uninfected CD4^+^ T cells and induce their apoptosis. These exosomes selectively contain three key mRNAs involved in cell death and fatty acid metabolism whose corresponding miRNAs are preferentially retained in the infected cells of origin: *aatk, slc7a1*, and *cdkal* (64). EBOV VP40 can be transported within exosomes and can modulate the expression of the RNAi components, including Dicer, Drosha, and Ago1 in recipient immune cells, ultimately resulting in cell apoptosis. VP40 has been suggested to be responsible for the suppression of the T cell and myeloid arms of the immune system, resulting in the virus being able to replicate in an immunocompromised host (19). Lastly, Ahmed and his team observed that exosomes from EBV-infected cells can induce B-cell and T-cell apoptosis through the Fas ligand-mediated extrinsic pathway (65). In summary, immune cell apoptosis can be induced by exosomes containing RNAs or proteins associated with the pathogens.

### Exosomes and the Inhibition of Infection

Exosomes play an important role not only in the process of infection by pathogens but also in anti-infection. Indeed, a range of responses are laid out to ward off the infection after pathogen invasions.

#### Inhibiting Pathogen Proliferation and Transmission Directly

Exosomes could participate in the fight against infections by restraining the proliferation and transmission of pathogens and especially of viruses. In fact, exosomes can prevent viruses from replicating and transcribing. Exosomes from healthy semen block the spread of HIV-1 from vaginal epithelial cells to target cells as well as the passage of HIV-1 through the vaginal epithelial barrier. Upon internalization of exosomes into vaginal epithelial cells, functional mRNA encoding APOBEC3G was transferred, making a potential connection between semen exosomes and the impairment of viral RNA reverse transcriptional activity. Semen-derived exosomes are, thus, found to decrease the intravaginal replication of the AIDS virus in mice as well as the virus systemic spread and viremia (66). In a parallel approach, exosomes from human vaginal secretions showed some inhibitory effects on HIV-1 infection. The exosomal fraction from this fluid decreased the efficiency of transmission and of reverse transcription and integration of HIV-1 vectors (67). Regarding HCV, some small RNAs (namely let-7f, miR-145, miR-199a, and miR-221) can target specific cellular factors or directly bind to the viral genome in order to block HCV replication. These miRNAs are transported *via* exosomes, which, therefore, mediate the antiviral process (68). During infection of cultured intestinal epithelium cells with a protozoan parasite, *Cryptosporidium parvum*, as well as following the stimulation of biliary epithelial cells with LPS, an activation of TLR4 signaling leads to the enhanced release of exosomes from these cells through the SNAP23-associated process of vesicular exocytosis. These exosomes transport epithelial antimicrobial peptides, which were found to bind to and decrease the viability and infectivity of *C. parvum* sporozoites (69). HCV replication can be controlled by exosomes derived from primary human liver sinusoidal endothelial cells that were able to stimulate type Ι or type ΙΙΙ IFNs (70). The delivery of anti-HCV factors to hepatocytes has been found to occur through exosomes released from TLR3-activated macrophages. Such exosomes contain miRNA-29 family members which either activate a cellular anti-HCV response or directly target HCV gene expression, thus inhibiting HCV replication (71). An antiviral activity of exosomes has also been observed in animals. Chicken biliary exosomes can inhibit the replication of avian leucosis virus subgroup J (72).

#### Inhibiting Infection by Stimulating Immune Responses

When pathogens enter the human body, immune responses are triggered, with the release of cytokines and the development of humoral and cellular immunity. The immune response mainly consists of three stages: induction (sensitization stage), proliferation and differentiation (reactive stage) and, finally, definition. Exosomes play an important role by the following.

##### Improving Monocyte-Macrophage Functions

Macrophages take part in specific and non-specific immunity. They can kill and clear pathogens nonspecifically after phagocytosis as well as mediate inflammatory responses. In a specific manner, macrophages act as immune-regulators and antigen-presenting cells. Their function in relation with innate immunity depends on the interaction of pattern recognition receptors such as TLRs, C-type lectin receptors and scavenger receptors with molecules on the surface of target organisms. Exosomes play a role in this process, as indicated by the observation that EVs of bacterial origin released from cells infected with m.tb can trigger TLR2 in uninfected macrophages and, as a consequence, result in cytokine responses (73).

Antigen presentation is crucial for immune responses. It is performed by antigen-presenting cells (APCs), also known as accessory cells, which can be divided into two types: MHC Ι and MHC ΙΙ. MHC molecules present peptides to other immune cells in order to mount an adaptive immune response. Macrophages can act as APCs. During infection pathogenesis, macrophages acquire the M1 (classically activated) or M2 (alternatively activated) phenotypes regarding their activation programs, and this depends on the microenvironment where they are. Since exosomes participate in establishing tissue microenvironments upon their release by different cell types, they play a role concerning macrophage differentiation and polarization.

Exosomes secreted by HCV-infected hepatocytes contain HCV ss-RNA associated with miR-122 and Ago-2, which can be taken up by circulating monocytes. In turn, these monocytes differentiate into M2 macrophages that express pro-inflammatory cytokines and collagen, increasing inflammation in the liver and leading to fibrosis (74). Exosomes released from *M.tb*-infected cells transported mycobacterial lipids and proteins like mannose-capped lipoarabinomannan, PIM and trehalose dimycolate to naïve macrophages, resulting in macrophage recruitment. As the recruited macrophages differentiate into multinucleated giant cells and epithelioid macrophages, the phase of rapid bacterial multiplication tended to be walled off by them, thus forming granuloma and controlling the containment locally at the level of granuloma (18). In another study, exosomes shed from *M. avium* sp. *paratuberculosis*-infected cells promoted the expression of CD80 and CD86 and the secretion of TNF-α and IFN-γ by macrophages, suggesting that exosomes from infected macrophages can be carriers of bacterial antigens and/or molecules that can induce an immune response in resting cells (75).

##### Promoting the Function of NK-Cells

NK cells are important effectors of the innate immunity and act as cytotoxic lymphocytes in peripheral blood. NK-cell function can be indirectly enhanced by exosomes in a different fashion from that of macrophage activation. HCV is a positive-stranded RNA virus that targets hepatocytes. Besides infection, intercellular transfer of HCV-RNA occurs by an exosome-mediated process. Exosomes from HCV-infected hepatocytes containing HCV-RNA fragments can be recognized by TLR3 in DCs, which then mature to express NK-activating ligands. At the same time, these exosomes evoke major cellular effectors and type Ι/ΙΙΙ IFNs in DCs, which facilitate NK induction against HCV (76). In another instance, EVs released from HBV-infected hepatocytes were found to contain viral nucleic acids and induce natural-killer group 2, member D (NKG2D) ligand expression in hepatic F4/80+ cells. NKG2D ligands trigger IFN-γ generation from NK cells. Furthermore, depleting exosomes from EVs markedly reduces the expression of NKG2D ligand, suggesting that exosomes play a role in NK cell activation (61).

##### Promoting the Function of T Cells

T cells play a key role in specific immune responses regarding both humoral and cellular immunity. Exosomes can induce T-cell functions by promoting the maturation of T cells and enhancing the expression of inflammatory cytokines.

The fact that macrophage- and DC-derived exosomes present MHC Ι and ΙΙ as well as T-cell co-stimulatory molecules on their surface strongly suggests that they may constitute an important element of antigen presentation mechanisms. Three models have been currently proposed for exosome-mediated antigen delivery to T cells: cross-dressing pattern, cross-presentation pattern, and direct exosome-induced T-cell activation (77).

In the cross-dressing pattern, exosomes from the infected cells including APCs could transfer preformed peptide-MHC complexes to the surface of the uninfected APCs, which could then present these antigens without having first phagocytosed an antigen-carrying organism nor having processed the antigen (78). For example, CD8^+^ dendritic cells incubated with LPS and an antigenic peptide can secret exosomes containing preformed peptide–MHC complexes. These exosomes can then be captured by paraformaldehyde-fixed DCs, i.e., DCs unable to reprocess antigens, and induce T-cell activation determined as an upregulation of CD69^+^ (79). An interesting study shows that DC-derived exosomes can acquire TLR ligands from bacteria and act alerting the immune system by activating bystander DCs. As a consequence, both the expression of TNFα and pro-inflammatory cytokine secretion by those cells are upregulated, and interaction with NKs results in an enhanced IFNγ secretion mediating enhanced Th1 polarization (80). Exosomes can actually contribute to generate a proper T-cell response other than direct presentation by macrophages and DCs. In an extreme case of exosome biogenesis impairment, Rab27a-deficient mice were incapable of trafficking mycobacterial components to exosomes. EVs isolated from *M.tb*-infected Rab27a-deficient mice showed a reduced capacity to elicit a pro-inflammatory response. Instead, exosomes from BCG-infected macrophages can promote a T-cell response by antigen cross-presentation (81). Chicken biliary exosomes were observed to influence immune responses by stimulating the proliferation of CD4^+^ and CD8^+^ T cells and monocytes from liver. They also inhibited avian leucosis virus subgroup J, which is an oncogenic retrovirus, from replicating in the DF-1 cell line (72). As for direct exosome-induced T-cell activation, Hwang and his colleagues found that APCs secret exosome-like vesicles which express ICAM-1 and B7 on their membrane as well as antigen-presenting peptide–MHC class I complexes, and which bind with CD8^+^ T cells to activate their proliferation and differentiation into peptide-specific effector cells (82).

In addition to participating in the antigen-presentation process, exosomes can also transport a variety of cytokines that promote T-cell activation and function. As an example, human peripheral CD3^+^ T cells activated with anti-CD3 and IL-2 release exosomes carrying large amounts of CCL5 (RANTES) that stimulate cytokine secretion and cause the proliferation of CD8^+^ T cells *in vitro*. Therefore, it can be speculated that these exosomes favor a cytotoxic response within an anti-infection process (83).

Exosomes can also affect T lymphocyte functions. An analysis of the impact of miR-155 loaded in *H. pylori*-infected macrophage-derived exosomes showed that the expression of inflammatory cytokines, including TNF-a, IL-6, and IL-23 is enhanced (84). In another study, outer membrane vesicles produced by Gram-negative bacteria were seen to act as vehicles that deliver LPS to the host cell cytosol. This results in enhanced cell death and the activation of IL-1 cytokines (28). In brief, exosomes are able to enhance T-cell functions by delivering cytokines.

##### Promoting the Function of B Cells

B stem cells develop from hematopoietic precursor cells in an ordered maturation and selection process. B cells and the antibodies they produce are the central elements of humoral immunity. As part of the adaptive immune system, B cells provide protection against an almost limitless variety of pathogens. Exosomes can specifically enhance B cell-mediated immune responses. For instance, exosomes from *Mycoplasma*-infected tumor cells can induce the generation of cytokines by splenocytes (85).

**Figure 3:**
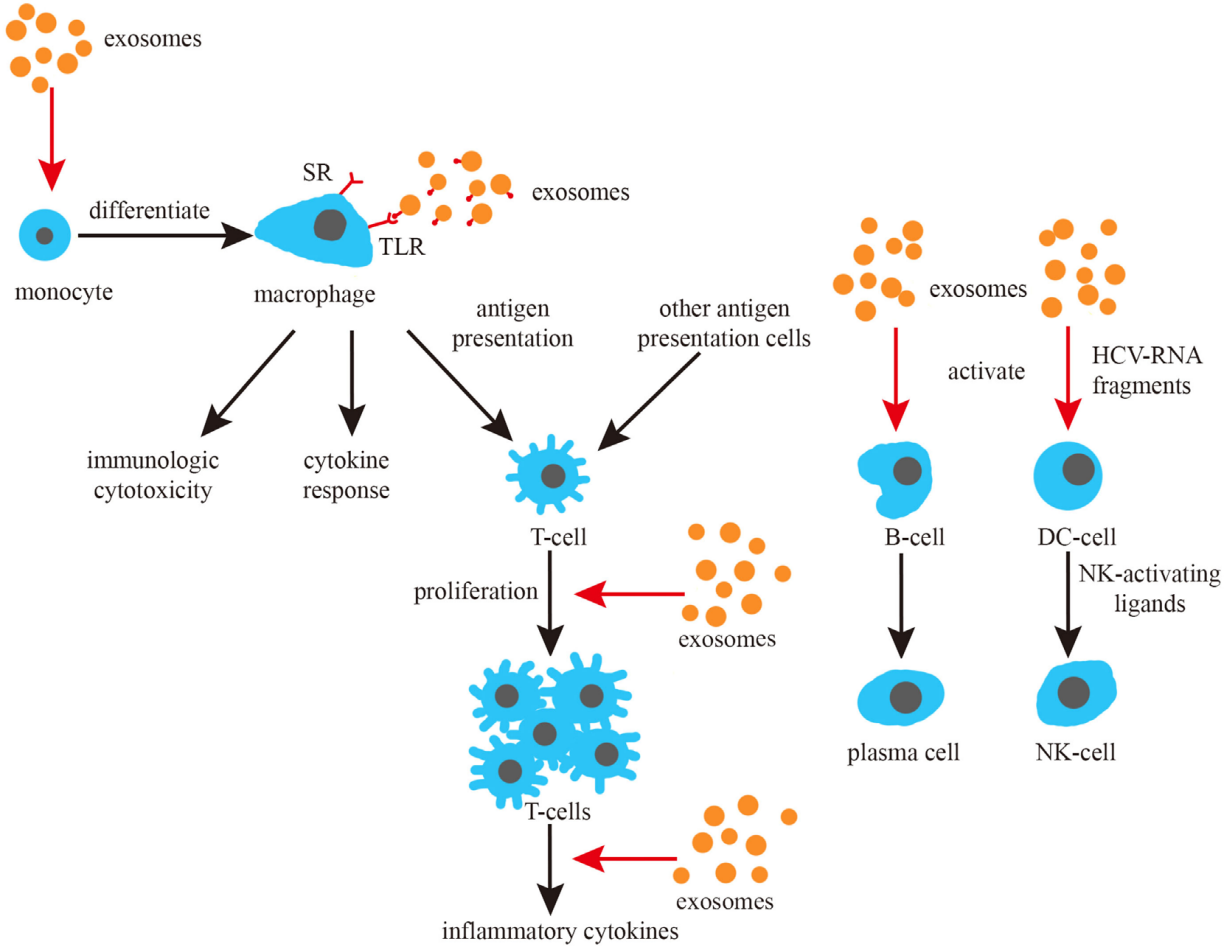
Exosomes and the induction of immune responses. Exosomes promote the differentiation of monocytes, can activate macrophage toll-like receptor (TLR) receptors and promote the cytokine response of macrophages. Exosomes can also promote the proliferation and cytokine response of T cells, as well as activate B and NK cells.

In brief, numerous studies from different perspectives have indicated that exosomes play a significant role in the fight against infections from different perspectives (Figure 3).

## Applications of Exosomes Regarding Pathogen Infections

As we have discussed, exosomes are involved in multiple steps during infections and the fight against them. They participate in the formation/modification of infection loci, the discrimination of antigens during the initial stages of infection, the regulation of cell apoptosis, and the modulation of immune cell functions. Exosomes also act as a source of antigens for the activation of T and B cells. As a consequence, the role of exosomes as potential therapeutic agents is being actively considered.

### Diagnosis of Pathogen Infection

Exosomes can be used as sources of body fluid biomarkers. They have been isolated from serum (86), bronchoalveolar lavages (87), urine (88), saliva (89), and others. Quantitative and qualitative differences in the composition of exosomes in health and disease have been reported (90). These differences, together with an easy isolation, make exosomes excellent biomarker reservoirs as well as potentially useful for diagnosis. Britton et al. demonstrated that the secretion of specific miRNAs within EVs from parasitic helminths can be the basis to develop novel and sensitive diagnostic markers of infection (91). Saá et al. found that a fraction of the transmissible spongiform encephalopathy-associated prion protein (PrPTSE) which is found in circulating blood is actually localized in exosomes isolated from plasma. This opens new avenues for further TSE research. Since exosomes are known to participate in cell-to-cell communications and are also able to cross the blood–brain barrier, the association of PrPTSE with exosomes may well serve to spread TSE from the periphery to the CNS (92).

### Therapy of Pathogen Infection

Exosomes can be useful as novel targets to develop new drugs. For example, it has been observed that *Trypanosome* EVs can cause changes in the physical properties of cell membranes which lead to the phagocytosis of erythrocytes, therefore, being a cause of anemia during acute trypanosomiasis. The possibility is then opened of developing inhibitors of the fusion of trypanosomal EVs with host cells which will reduce the likelihood of developing anemia (50). Researchers have shown that exosomes secreted from umbilical cord MSCs (Umsc) exhibit a potent anti-HCV activity by targeting the replication of the virus. The fact that a series of miRNAs (*let-7f, miR-145, miR-199a, and miR-221*) are specifically transported by exosomes illustrates a promising method of anti-HCV therapy (68). Exosomes loaded with Interferon-induced transmembrane protein 3 can transmit antiviral activities from one cell to another during dengue virus infection (93).

Exosomes can also transfer antiviral molecules, such as APOBEC3G from non-permissive liver non-parenchymal cells to permissive hepatocytes during HBV infection (94). In brief, new antiviral strategies could be developed with exosomes serving as nano shuttle vehicles for drug delivery.

Exosomes could act as vaccines to prevent infections. For instance, del Cacho et al. found that exosomes derived from dendritic cells infected with the parasite *Eimeria* convey protection in a poultry model (95). Research by Martin-Jaular et al. has indicated that exosomes derived from reticulocytes could be explored to develop vaccines against malaria infections (96). However, problems can be envisaged regarding the use of exosomes in therapies. As potential vaccines, the fact that exosomes contain various proteins as well as other molecules could represent a shortcoming. While exosomes no doubt are plastic entities, many additional clinical tests will be necessary to apply them in therapeutics. As research into exosomes deepens, new strategies are likely to emerge leading to valid approaches in the fight against infections.

## Conclusion

Exosomes are membrane-bound vesicles measuring 50–100 nm present within late endosomes and containing proteins and RNAs. They are secreted from both pathogens and host cells and can be used by both pathogens and hosts to affect and modulate infection processes. Exosomal vesicles can transmit signals between pathogens and hosts regarding various aspects of host defense.

**Figure 4:**
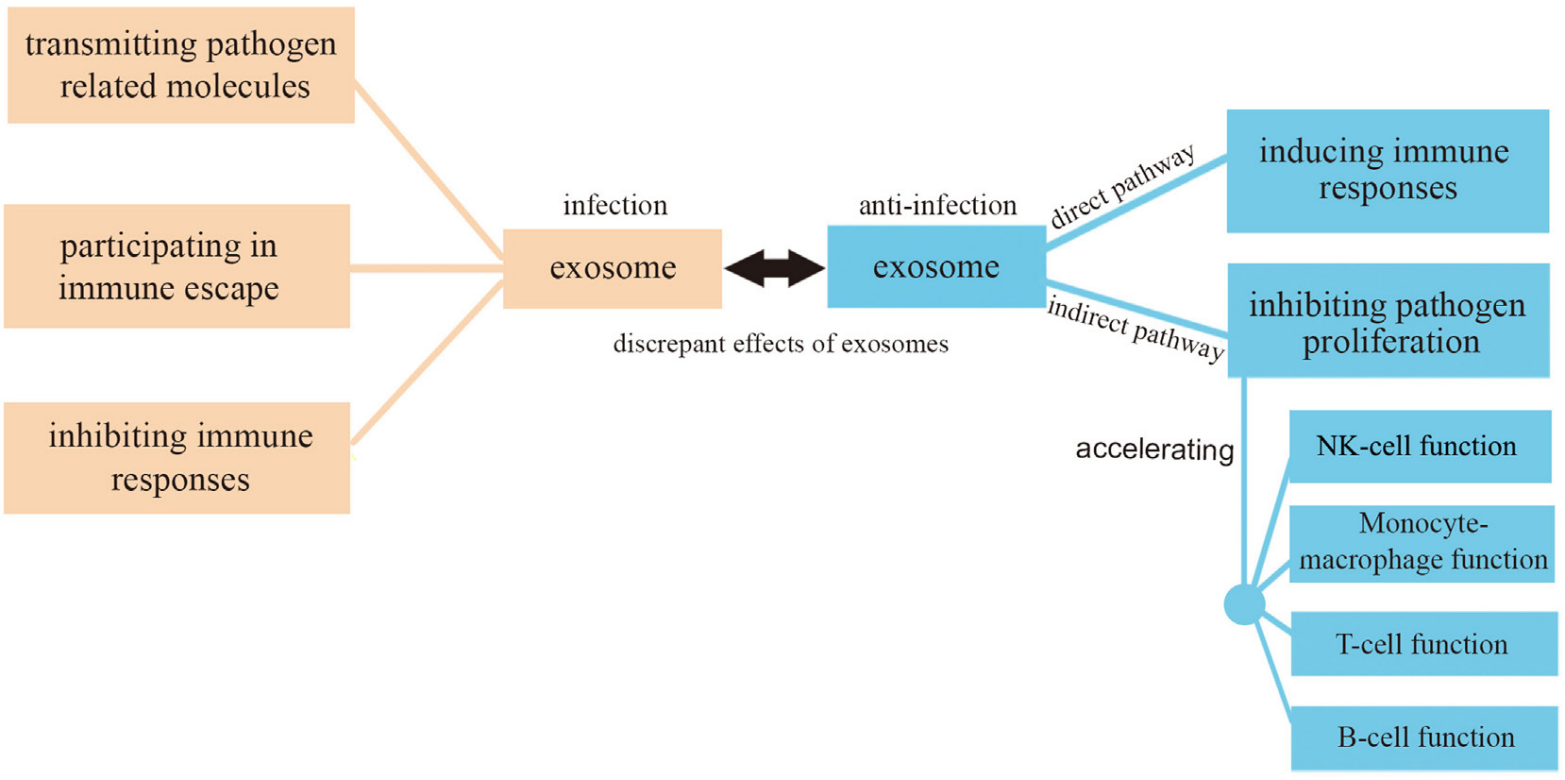
Exosomes in infection and anti-infection. Exosomes participate in both infection and anti-infection processes ranging from pathogen infection to the regulation of immune responses.

In this review, we have focused on exosome functions in relationship with infection and anti-infection. On the one hand, exosomes derived from pathogens containing pathogenic proteins and RNAs can promote infection in three ways: (1) causing further infection by transmitting pathogen-related molecules; (2) participating in pathogen immune escape mechanisms; (3) inhibiting immune responses by inducing immune cell apoptosis. On the other hand, exosomes can play anti-infective roles by (1) inhibiting pathogen proliferation and infection directly; (2) inducing immune responses including an enhancement of the function of monocyte-macrophages, NK cells, T cells, and B cells (Figure 4). There must be a balance between infection and anti-infection processes, with exosomes being crucial messengers modulating this balance in different ways, as discussed in the present review. A close connection exists between exosomes and the immune system which involves immune induction, evasion, and inhibition. Conversely, the immune system has an impact on exosomes, reinforcing the concept of a dual promoting-inhibiting function. It can be, therefore, speculated that exosomes constitute an important factor of the immune system with the potential to affect its delicate balance.

## Author Contributions

LT conceived and designed the work. WZ, XJ, JB, YW, HL, and LT discussed, wrote, and edited the manuscript. LT revised the manuscript. All authors read and approved the final manuscript.

## Conflict of Interest Statement

The authors declare that the research was conducted in the absence of any commercial or financial relationships that could be construed as a potential conflict of interest.
